# Re-Endothelialization of Decellularized Liver Scaffolds: A Step for Bioengineered Liver Transplantation

**DOI:** 10.3389/fbioe.2022.833163

**Published:** 2022-03-10

**Authors:** Kewei Li, Mohammad Tharwat, Ellen L. Larson, Philipp Felgendreff, Seyed M. Hosseiniasl, Anan Abu Rmilah, Khaled Safwat, Jeffrey J. Ross, Scott L. Nyberg

**Affiliations:** ^1^ Department of Surgery, Mayo Clinic, Rochester, MN, United States; ^2^ Department of Pediatric Surgery, West China Hospital of Sichuan University, Chengdu, China; ^3^ General Surgery Department, Faculty of Medicine, Zagazig University, Zagazig, Egypt; ^4^ Department for General, Visceral and Vascular Surgery, University Hospital Jena, Jena, Germany; ^5^ Miromatrix Medical Inc, Eden Prairie, MN, United States; ^6^ William J. von Liebig Center for Transplantation and Clinical Regeneration, Mayo Clinic, Rochester, MN, United States

**Keywords:** bioengineered livers (BELs), liver transplantation, decellularization, scaffolds, heterotopic transplantation, orthotopic transplantation

## Abstract

Bioengineered livers (BELs) are an attractive therapeutic alternative to address the donor organ shortage for liver transplantation. The goal of BELs technology aims at replacement or regeneration of the native human liver. A variety of approaches have been proposed for tissue engineering of transplantable livers; the current review will highlight the decellularization-recellularization approach to BELs. For example, vascular patency and appropriate cell distribution and expansion are critical components in the production of successful BELs. Proper solutions to these components of BELs have challenged its development. Several strategies, such as heparin immobilization, heparin-gelatin, REDV peptide, and anti-CD31 aptamer have been developed to extend the vascular patency of revascularized bioengineered livers (rBELs). Other novel methods have been developed to enhance cell seeding of parenchymal cells and to increase graft functionality during both bench and *in vivo* perfusion. These enhanced methods have been associated with up to 15 days of survival in large animal (porcine) models of heterotopic transplantation but have not yet permitted extended survival after implantation of BELs in the orthotopic position. This review will highlight both the remaining challenges and the potential for clinical application of functional bioengineered grafts.

**GRAPHICAL ABSTRACT:**
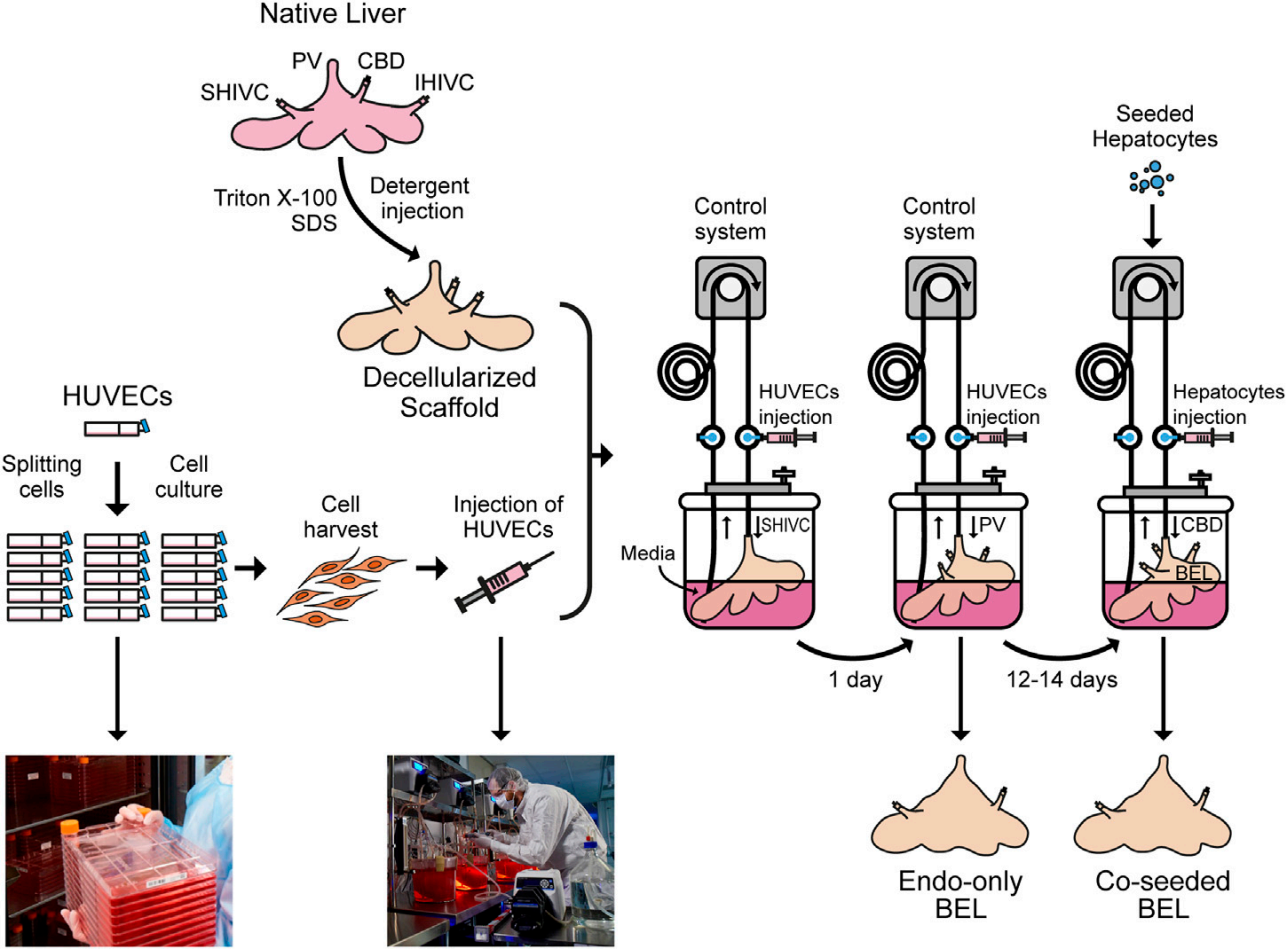
Native porcine livers are cannulated on the PV, IVC and SVC and perfused with Triton X-100 and SDS solutions to get a decellularized liver. Meanwhile, HUVECs are cultured in 2D tissue culture flasks, harvested and injected through the SHIVC of the decellularized liver, followed by the PV 24 h later to get endo-only BEL. After 12–14 days in media the seeded hepatocytes can be injected through the CBD to get co-seeded BEL.

## Introduction

Chronic liver disease is a major global health challenge, responsible for approximately two million deaths each year (Melaram, 2021). As a result, liver disease accounts for 3.5% of all deaths worldwide. This evolution can be attributed to the rising prevalence of liver cirrhosis and hepatocellular carcinoma (Asrani et al., 2019). Despite the fact that the cause of this disease varies from country to country. Hepatitis B and C viruses are primarily responsible for the pathogenesis of cirrhosis and HCC in Asian countries, whereas toxic factors are mostly responsible for chronic liver disease in Western industrialized nations (Wang et al., 2014). Also, NAFLD and ALD are on the rise, notwithstanding the efforts to eradicate and control viral hepatitis through programs such as WHO 2030. Patients with liver disease are becoming an increasingly difficult medical and socioeconomic challenge, as this trend is unlikely to be reversed in the foreseeable future. Furthermore, we need to improve and expand our therapeutic options to ensure that patients are provided the opportunity for treatment (Paik et al., 2020).

Currently, only a limited number of treatment options are available for patients with end-stage liver disease (ESLD). Most of these options aim to save the liver quality and stabilize the disease with a palliative intent. The only curative treatment for patients with ESLD is liver transplantation. This invasive procedure offers the opportunity not only to replace the entire affected organ, including the local tumor, but also to prevent additional metastases (Jimenez-Romero et al., 2019; Yang et al., 2019; Mazzaferro et al., 2020). The key proof for the clinical effectiveness of curative liver transplantation is the patients’ postoperative survival. As a result, the 1- and 5-years survival rates of ESLD patients following transplantation were generally 70–90% and 60–80%, respectively (Haydon and Neuberger, 2000). To optimize the likelihood of success of liver transplantation, it is critical to identify appropriate recipients and make transplantable donor organs readily available. One of the most significant obstacles to modern transplantation surgery is the scarcity of donor organs acceptable for transplantation, as well as the related higher mortality of patients on the waiting list (Arulraj and Neuberger, 2011).

Xenogenic liver transplantation offers an infinite supply of donor organ material. However, numerous significant translational difficulties, such as immunological incompatibility, basic variations in basal metabolism between humans and other animals, and coagulopathy, stand in the way of this solution (Mazza et al., 2018; Suchy et al., 2018). Alternatively, as a result of low cell engraftment effectiveness and loss of cell function over time, isolated hepatocyte transplantation as an alternative to whole liver transplantation may only be considered as interim metabolic treatment of inherited liver disease or a bridge therapy in acute liver failure as it does not correct the sequelae of portal hypertension created by a cirrhotic liver (Nicolas et al., 2016). Consequently, it is essential to find new and innovative sources for usable organs as long as others substitutes such as xenogenic liver transplantation and isolated hepatocyte transplantation failed to compensate the need for transplantable organs. One experimental approach that has been advanced by many transplantation experts is the development of bioengineered livers as an alternative and potentially inexhaustible organ source (Pla-Palacin et al., 2018; Mirdamadi et al., 2020).

BEL is a promising therapeutic alternative to liver transplantation, aiming to replace or regenerate the human liver. It is emerging as one of the most significant scientific advances in the field of tissue engineering (Gilpin and Yang, 2017; Li et al., 2017; Agarwal et al., 2019; Kakabadze et al., 2019; Naeem et al., 2019). Moreover, this technique may potentially offer an endless source of patient-specific livers as it could eliminate the need for immunosuppression of recipients (Croce et al., 2019).

## Principals for Bioengineered Livers

In order to develop a completely competent transplantable organ utilizing BEL, the two stages of decellularization and recellularization are necessary (Badylak et al., 2011; Baptista et al., 2011). Decellularization seeks to create a biocompatible decellularized scaffold that preserves the three-dimensional architecture and biochemical content of the original tissue while removing the cellular material to decrease the immunological reactivity (Uygun et al., 2010; Debnath et al., 2020; Hou et al., 2020; Moulisova et al., 2020). Enzymes and chemicals, along with mechanical agitation, freezing/thawing, and sonication, are various strategies for decellularization by disturbing cells and liberating their substance. In any case, the utilization of substances such as nonionic Triton X-100 and ionic sodium dodecyl sulfate (SDS), thought to be limited at whatever point conceivable to stay away from harm to the microarchitecture of the original ECM (Badylak et al., 2011). Decellularization may result in the loss of ECM components as well as permanent damage to the microarchitecture of the ECM. The success of decellularization is better evaluated by a multi-scale quantitative method based on measuring the remaining DNA and on histological evaluation of the preserved liver-specific microarchitecture (Moulisova et al., 2020).

ECM-based scaffold components and functions are assumed to be relatively conserved across species, and thus the scaffolds are thought to be less or nonimmunogenic, even when obtained from xenogeneic sources (Wang et al., 2011; Badylak, 2014; Struecker et al., 2015). The creation of a biological scaffold is closely tied to the success of liver regeneration, in addition to providing structural stability of the organ. The scaffold is critical as a substrate for cell adhesion, differentiation, and proliferation, as well as for facilitating vascularization for nutrition, oxygenation, and the physiological discharge of waste cell-derived metabolites (Ohashi et al., 2007; Haycock, 2011; Park et al., 2016; Naeem et al., 2019). Put another way, the ECM-based scaffold is no longer just considered as a lifeless or barren environment such as 2D cultures on Petri dishes, but rather a platform that actively participates in the dynamic control of the biological activity of the liver *in vivo* through creating a similar environment to the native one. This environment encourages cell growth and proliferation, preserves the complex structure of the vascular tree and facilitates cell-cell interaction (Ansari et al., 2020).

Two primary processes are regarded to ensure a proper recellularization of a decellularized graft: cell seeding followed by perfusion culture, which is commonly used to prepare cells for *in vivo* activity. The objective of cell seeding is to distribute cells with the graft matrix using a physiological composition and an appropriate number of cells similar to that in the native liver. For example, the adult human liver contains around 2.8 × 10^11^ hepatocytes (Baptista et al., 2011). Furthermore, the arrangement of cells into niches inside the scaffold is essential to closely approximate their normal distribution. Whether parenchymatous cells, such as hepatocytes which are responsible for function, or non-parenchymatous cells, such as endothelial cells which serve as an antithrombotic barrier, the distribution of these cells will determine the functionality of the new engineered liver graft. These cells are introduced into the scaffold by a variety of techniques, including injection and perfusion (Badylak et al., 2011; Baptista et al., 2011).

Significant progress has been made in the fabrication of tissue-engineered structures such as blood vessels, urinary bladder, and trachea. The transplantation of these structures requires the anastomosis of smaller vessels to the recipient circulation. However, a larger and more complex vascular supply will be necessary to maintain patency and perfusion of the network of vessels required for functionality of a totally tissue-engineered solid organ such as the heart, lung, and liver (Badylak et al., 2011; Baptista et al., 2011; Bao et al., 2015).

The liver is a particularly active metabolic organ, which presents prospective obstacles for the development of clinical-scale, implantable organs with significant perfusion and vascularization requirements (Zheng and Roberts, 2016). For example, normal function of hepatocytes requires a complex hierarchical vascular network that includes multiple enormous branches at the organ size as well as complex microvessels at the sinusoidal level (Shirakigawa et al., 2013; Watanabe et al., 2019). Furthermore, hepatocytes are rich in mitochondria and consume 10-fold more oxygen than most other cells of the body. Due to the high oxygen requirement of hepatocytes, the liver’s normal vascular anatomy provides transport of oxygen and nutrients to approximately 200 μm or less. Similar requirements are necessary to avoid ischemic injury in BELs after transplantation (Cho et al., 2007; Uygun et al., 2010).

## Early Stages of Decellularization and Revascularized Scaffolds

One of the first studies of a tissue engineered organ was reported by Ott and colleagues in 2007, after they had developed a decellularization technique on rat hearts by coronary perfusion with detergents including SDS and Triton-X100. These methods succeeded in disrupting cells and nuclei and eliminating DNA without significant damage to the extracellular matrix scaffold. After recellularization, the new construct successfully maintained contractile function and drug responsiveness after 8 days in culture (Ott et al., 2008). These results represented a significant breakthrough for the application of this technique to other organs. Namely, an efficient and reproducible method yielding a natural acellular three-dimensional framework void of cells from any organ (Croce et al., 2019).

This technique was subsequently optimized for liver decellularization, then preliminarily expanded with the development of methods for re-establishing a vascularized network within the decellularized graft using endothelial cells (ECs). Thus, the keystone for successful BEL is the presence of an intact vascular tree for optimal reperfusion (Uygun et al., 2010; Baptista et al., 2011; Struecker et al., 2015). That’s why Baptista and colleagues designed their decellularized rat liver grafts to ensure getting an intact capillary bed with indefinite leakage *in vitro*. Also, they transplanted the grafts into the abdominal cavity of rats. In heparinized host animals, these grafts maintained normal blood flow for up to 1 hour (Baptista et al., 2011).

Uygun and colleagues demonstrated an approach to generate decellularized and recellularized liver grafts which could support liver-specific function, including albumin secretion, urea synthesis, and cytochrome P450 expression. This method introduced primary rat hepatocytes *via* the portal vein of a decellularized scaffold. After auxiliary liver grafts were transplanted into rats and kept for 8 h, the TUNEL staining of those harvested grafts revealed minimal ischemic damage. Preliminary revascularization with ECs also showed attachment and viability within the graft *in vitro* (Uygun et al., 2010). Also, Baptista and colleagues populated their scaffolds with human fetal liver and endothelial cells, resulting in a liver-like tissue *in vitro*. Endothelial cells were implanted throughout the whole circle of a vascular channel by using the vena cava and portal pathways, and the cells displayed preserved cell-cell connections. They also discovered that the adherence and presence of platelets were significantly reduced in endothelialized scaffolds (Baptista et al., 2011). This finding that coverage of the vascular tree by endothelial cells was significant for preventing platelet adherence and maintaining vascular patency suggested that synthetic grafts could be implanted without drug anticoagulation (Ko et al., 2015). However, until a method of complete endothelial seeding of perfused grafts was optimized, blood clotting of grafts remained problematic and handicapped the progress of BELs (Orlando et al., 2012; Kojima et al., 2018).

## Passivation Methods and Anticoagulants for Revascularized Bioengineered Livers (rBELs)

Other considerations are important to maintain graft patency. For example, collagenous fibers from the vascular basement membrane that are exposed to blood flow activate the clotting cascade during extracorporeal blood perfusion or after *in vivo* transplantation (Kipshidze et al., 2004; Caralt et al., 2014; Wang et al., 2020). The surface charge of graft materials is a pivotal reason why grafts are prone to thrombosis. Heparin has a large negative charge density and repels albumin and fibrinogen, preventing them from sticking to the graft due to static repulsion between heparin and these proteins. The issue is that heparin release patterns are frequently characterized by a substantial initial burst of heparin. After which very little or nothing remains on the scaffold, so strategies to limit and control heparin release have been developed “Heparin immobilization” (Matsuzaki et al., 2021). Therefore, to get better hemocompatibility after *in vivo* perfusion of decellularized scaffolds of swine liver, heparin immobilization technique was used by Bao et al. (2015) before auxiliary transplantation. In their trial, they used the swine median lobe scaffolds where the PV of the graft was anastomosed to the host left renal vein as an inflow and the SHIVC of the graft was anastomosed to the host splenic vein as an outflow. It is found that heparin immobilized on the scaffolds can release slowly, therefore achieving improved anticoagulation. The heparinized scaffolds showed convenient blood flow for 60 min, compared to no blood flow throughout the control scaffolds after 20 min of perfusion. *In vitro* studies indicated the heparinized scaffolds could support rat primary hepatocytes and human umbilical vein endothelial cells (HUVECs) adhesion and growth (Bao et al., 2015).

Hussein et al. (2016) found that a heparin-gelatin mixture (HG) improved ECs’ ability to migrate and adhere to vessel discs, which could efficiently cover the vascular network lumen and maintain function and proliferate. Gelatin contains many integrin binding sites for EC adhesion, migration, and differentiation, similar to the binding sites that are detected in natural collagen and other extracellular matrix proteins (Hajiali et al., 2011; Hussein et al., 2016). The addition of heparin to gelatin increases cell recruitment and migration, which is due to its highly negative charge density and electrostatic interaction with positively charged proteins in the ECM (Trindade et al., 2008; Camci-Unal et al., 2013; Hussein et al., 2016). The authors manufactured bioengineered scaffolds based on the decellularized right lateral lobes of swine livers, which were co-cultured by hepatocellular carcinoma cells (HepG2) and ECs (EA.hy926 cells) and then heterotopically transplanted into pigs. Some of these scaffolds were HG-coated before ECs perfusion. After 1 h of *in vivo* implantation, the scaffolds were retrieved, canulated for portography, and tissue specimens were taken to assess HepG2 cells function. Vascular patency was demonstrated in HG-precoated scaffolds, and their HepG2 cells exhibited greater metabolic and synthetic function than those of uncoated scaffolds. Moreover, the HG-precoated re-endothelialized grafts revealed no thrombosis after 24 h of *ex vivo* whole-blood perfusion (Hussein et al., 2016).

While heparin immobilization scaffolds were promising as engineered liver grafts, this process was only temporary and replenishment of the immobilized heparin was not possible *in vivo* (Baptista et al., 2016; Meng et al., 2019). Accordingly, reendothelialization appeared to be necessary to provide bioengineered livers with persistent anti-thrombotic capability (Meng et al., 2019). Meng et al. (2019) reported that gelatin hydrogel-based perfusion significantly increased the number of immortalized endothelial cells that were retained in the decellularized rat liver scaffolds, within which active blood flow could be observed 8 days post-transplantation (Meng et al., 2019).

Substances such as polyethylene glycol, zwitterionic polymers, heparin or thrombomodulin are used to coat the ECM in order to achieve surface modification of it. While this surface modification efficiently reinforced hemocompatibility in the liver scaffolds, these modified surfaces appeared too hydrophilic to facilitate ECs adhesion to the vessel walls. Devalliere et al. (2018) presented an efficient approach for improving revascularization of acellular liver scaffolds by conjugating the REDV cell-binding domain *in vitro*. REDV-ELP peptide conjugation facilitates cell-specific binding of ECs, leading to the formation of a uniform and well-spread EC monolayer, significantly reducing activation and adhesion of platelets to the vasculature without functionality change or toxicity in hepatocytes. As opposed to surface coating techniques, REVD-ELP peptide provides an additional cell adhesion signal to the ECM of the liver scaffolds that improves ECs attachment (higher level of VE-cadherin) and proliferation (higher level of VEGF), as well as maintains the structural and mechanical integrity of ECM (Devalliere et al., 2018).

In general, passivation methods and anticoagulants such as heparin immobilization, heparin-gelatin and REDV peptide have been attempted to increase the vascular patency of BELs and increase the survival of the graft by keeping them well perfused without ischemic damage. However, reported survival of liver grafts prepared by passivation methods has not exceeded 8 days (Bao et al., 2015; Hussein et al., 2016; Park et al., 2016; Saleh et al., 2020; Kim et al., 2021).

## Anti-Endothelial Cell Antibodies and Aptamer for Revascularized Liver Scaffolds

Ko and colleagues demonstrated the generation of clinically relevant-sized ECM scaffolds with an intact vascular network extending to the capillary bed in a porcine model. To enhance the attachment of vascular endothelial cells expressing GFP protein (MS1) under physiologic blood flow, they developed an effective method by conjugating anti-endothelial cell antibodies (rat anti-mouse CD31 antibody). They heterotopically transplanted the revascularized liver scaffolds with inflow from the renal artery and outflow to the renal vein; the transplants were able to withstand physiologic blood pressure and maintain blood flow for 24 h (Ko et al., 2015).

Although the anti-CD31 antibody ensures better attachment of endothelial cells, in regard of the limitations of mass-producing ability and immunogenicity, Kim and colleagues adopted an anti-CD31 single-stranded DNA aptamer as a coating agent in the context of re-endothelization scaffolds. Compared to antibodies, aptamers have lower immunogenicity and stronger binding affinity for particular proteins (Song et al., 2012; Toh et al., 2015; Zhou and Rossi, 2017; Kim et al., 2021). Next, the aptamer-coated scaffolds were repopulated with HepG2 cells, LX2 cells, HUVECs, and MSCs (Kim et al., 2021). Quantitatively, Kim and colleagues achieved higher endothelial coverage (95.53 ± 3.1%) and more re-endothelialized vessels per field (87.37 ± 1.59%) in the aptamer group compared to constructs without aptamer coating. They perfused the scaffolds through the rat renal circulatory system for 2 h. After perfusion with whole blood, the aptamer-coated constructs had low levels of integrin aIIb-positive platelets (a marker expressed by activated platelets) by immunostaining analysis, whereas a number of integrin aIIb-positive platelets accumulated around the vascular network in the anti-CD31 antibody-coated scaffolds. Furthermore, they also showed that aptamer-coated scaffolds, when implanted into the interlobular space of the cirrhotic liver, could ameliorate hepatic cirrhosis and restore liver function in the TAA-induced hepatic fibrosis rat models (Kim et al., 2021).

## iPSCs-Derived Cells in Bioengineered Livers

Even with advancements in immunosuppressive regimens for transplant patients, the requirement for long-term medication with systemic adverse effects, as well as being an immunocompromised patient, remain significant hurdles for organ transplantation. One of the long-term aims of BEL transplantation is to eliminate the need for immunosuppressor drugs by producing donor-matched grafts that do not trigger the immune system, resulting in graft rejection (Wadström et al., 2017). Induced pluripotent stem cells (iPSCs) from humans are a readily available source of reprogrammed adult somatic cells which would participate in producing donor-matched grafts. Many experiments were conducted in order to create hepatocyte-like cells obtained from iPSCs (Bizzaro et al., 2019). Park and colleagues reported the first attempt to repopulate decellularized rat liver scaffolds with functional iPSC-Heps. When the recellularized liver scaffolds were heterotopically transplanted into rats for up to 2 h, the grafts expressed hepatocyte markers (AFP and ALB) and did not rupture. However, coagulation occurred in all grafts after about one to 2 h of *in vivo* blood perfusion (Park et al., 2016). Also, it was suggested that iPSCs-derived endothelial cells might be a potential substitute for reconstructing the vascular network in bioengineered organs as they could be recipient-specific and reduce or even remove the need for immunosuppression after transplantation (Clayton et al., 2015; Jiang et al., 2015; Park et al., 2016). However, it is found that iPSCs are immunogenic cells which can activate NK cells and trigger T cell-dependent immune response. This immunogenicity can be attributed to epigenetic abnormalities, coding mutations, or genes translocation which lead to generating immunogenic antigens or decreased expression of self-antigens. So, the immunogenicity of these cells should be examined before their clinical application (Zhao et al., 2011; Liu et al., 2017; Otsuka et al., 2020).

## Improved Revascularized Bioengineered Livers Survival in Heterotopic Porcine transplantation Model

Our team has shown that complete re-seeding of decellularized whole porcine livers with human umbilical vein endothelial cells (HUVECs) and implantation heterotopically into immunosuppressed pigs could sustain perfusion for up to 15 days. We identified glucose consumption rate (GCR) as a reliable metric for non-destructively monitoring cell proliferation and ultimately predicting graft patency following blood perfusion (Shaheen et al., 2020).

More recently, we studied the co-seeded BELs, produced by adding primary porcine hepatocytes after HUVEC reendothelialization, to evaluate the function of the grafts in heterotopic position. Graft viability was stable before and after the transplant. A portosystemic shunt was employed to limit portal hypertension due to the limited space and smaller graft size in this heterotopic transplantation model. These studies followed our stepwise approach to using larger grafts with greater functionality in an orthotopic model (Anderson et al., 2021).

## Updated Orthotopic Liver transplantation Data for Revascularized Bioengineered Livers

Heterotopic liver transplantation can be challenging in clinical practice due to a variety of factors, including suboptimal venous outflow, limitation of space for the graft, and poor long-term outcomes. Thus, heterotopic liver transplantation has remained a rare approach clinically compared to orthotopic liver transplantation (Anderson et al., 2021; Rammohan et al., 2021). Nyberg and colleagues have created an orthotopic liver transplantation model with rBELs. We performed the liver transplant by caval interposition technique as previously reported by Calne et al. (1968) rather than the piggy-back technique as there is no plane of dissection between the liver and IVC in pigs. In our preliminary functionality studies only anastomosis to the SHIVC, IHIVC, and PV were performed. The hepatic artery and common bile duct were not utilized. SHIVC was sewn by traditional end-to-end technique. However, end-to-end anastomoses to IHIVC and PV were performed over a silicone tube insertion method to minimize the anhepatic phase and the ischemic time of rBELs implantation.

**FIGURE 1:**
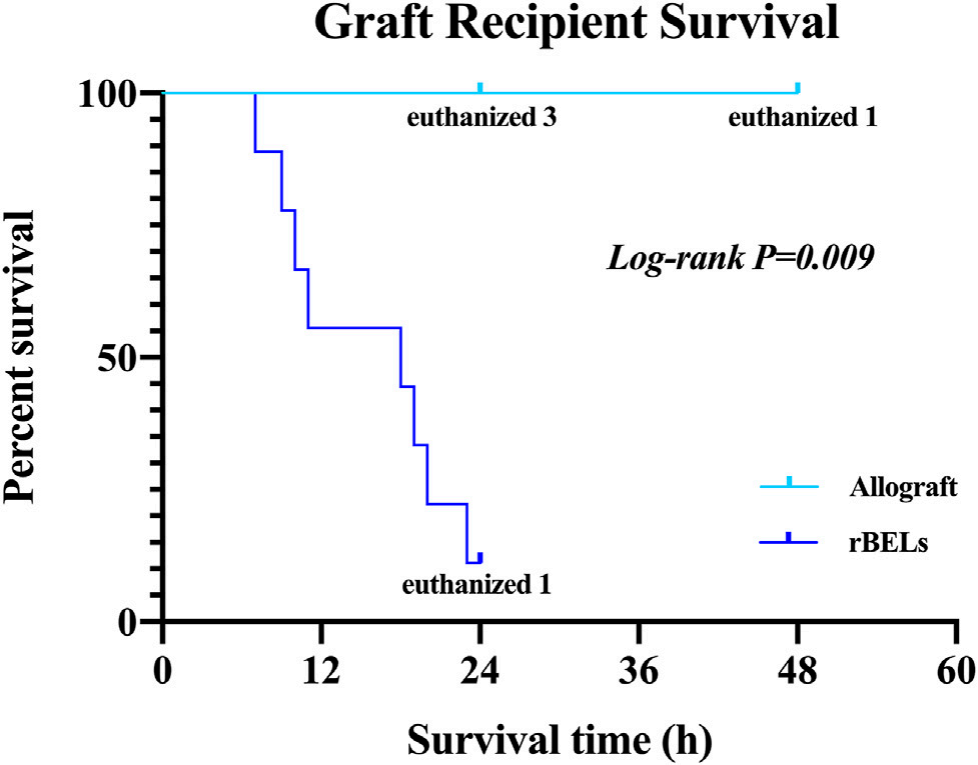
Kaplan-Meier analysis of survival of the allograft group and the rBELs group. Survival rate at 24 h was significantly higher in the allograft group compared to rBELs group (4 of 4, 100% vs. 1 of 9, 11.1%, *p* = 0.01). The study endpoint was death or the presence of a death equivalent endpoint. Death equivalent endpoints were: absent graft blood flow, grade IV hepatic encephalopathy, and uncontrolled bleeding, and animals were euthanized.

**FIGURE 2:**
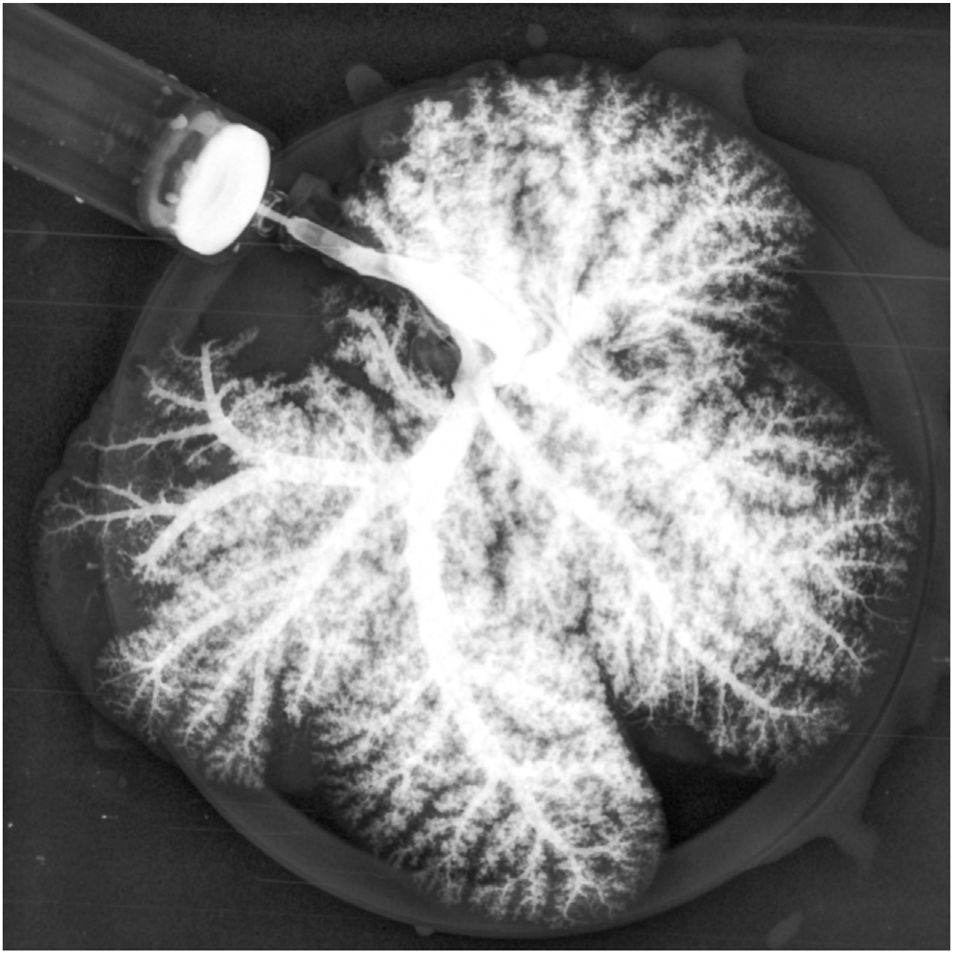
Portal venogram of Decell-recell liver graft demonstrating patency of vasculature and no evidence of thrombosis after 1-h *in vivo* perfusion.

As expected, the survival rate at 24 h was significantly higher in the pig allograft group compared to the rBELs group (4 of 4, 100% vs. 1 of 9, 11.1%, *p* = 0.01); the median survival time in the rBELs group was 18 h (range, 7–24 h) as illustrated in Figure 1. However, our rBELs showed patent vasculature with good graft reperfusion *in vitro* portal venogram and *in vivo* on declamping the PV, as shown in the Supplementary Videos. We also assessed the patency of the vasculature by portal venogram after 1 hour of *in vivo* perfusion with an intact vascular tree and no evidence of thrombosis (see Figure 2).

**FIGURE 3:**
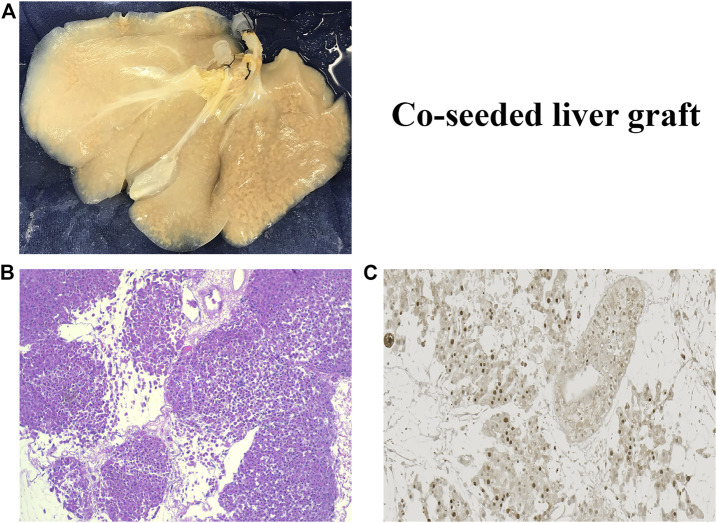
**(A)**. Decell-recell liver graft co-seeded with HUVEC endothelial cells and primary hepatocytes. **(B)**. Light microscopy of co-seeded graft showing lobular structure after reseeding (H&E staining). **(C)**. IHC microscopy of co-cultured graft showing mitotically active cells after reseeding (Ki67 staining).

The rBEL grafts shown in Figure 1 and Figure 2 were “endo-only grafts” seeded with HUVEC cells; they lacked hepatocytes and did not possess an intact biliary system required for clinical use. Liver grafts co-seeded with both HUVECs and primary hepatocytes have been studied on the bench and in heterotopic position, and soon may be ready for orthotopic transplantation studies. The co-seeded grafts showed an intact lobular structure after reseeding on H & E staining with mitotically active cells on staining with Ki-67 (see Figure 3). These data suggest steady progress of rBELs towards eventual clinical practice.

## Clinical Relevance of Revascularized Bioengineered Livers Data

The survival data shown in Figure 1 after transplantation of endo-only rBEL grafts may have clinical relevance to human surgeries. In particular, in some regions, it is standard to perform *ex vivo* liver resection and autotransplantation for end-stage hepatic alveolar echinococcosis. In the anhepatic phase of this human operation, the native liver is completely removed from the abdominal cavity for treatment, an artificial vascular graft is applied to temporarily reconstruct the IVC, and a portocaval shunt is established (Yang et al., 2018). The reported anhepatic phase in alveolar echinococcosis ranges from 104 to 879 min (Aji et al., 2018; Yang et al., 2018; Yang et al., 2020). According to the anhepatic phase of human reality viewpoint and comparing it with the survival data of our study, our rBELs would be efficient for sustaining physical perfusion phase in the absence of visible clots if they are used for this purpose in the future.

## Conclusion

The increasing need for liver transplantation as the only curative treatment for liver failure makes BEL a glimmer of hope for the future. Many obstacles stand in the way of achieving functional transplantable BELs, such as finding a suitable platform for the cells with proper dispersion and getting a patent vasculature. The use of scaffolds and the development of decell-recell techniques coupled with the availability of different cell lines and sources made a quantum leap in the development of BELs. The decellularized scaffolds became a platform that is not only a cell container but also a participant in their activity and functionality. Many trials were conducted to overcome the problem of blood clotting and failed reperfusion. Re-endothelialization is considered a cornerstone for a successful reperfusion phase. Not only by applying endothelial cells but also with proper attachment and extension throughout the vascular tree. Also, the use of anticoagulants, such as heparin immobilization, heparin-gelatin, and REDV peptide, has also been employed to improve BEL vascular patency. However, to the best of our knowledge, the grafts’ survival during *in vivo* perfusion did not surpass 8 days by any method. Shaheen et al. (2020) showed that sustained perfusion of our revascularized bioengineered livers heterotopically transplanted into immunosuppressed pigs survived for up to 15 days, which is considered a step in creating a more stable bioengineered graft. In our unpublished new study, we created an orthotopic BEL model that could survive for up to 24 h. However, as our rBELs are still preclinical trials, they are considered promising results as the 24-h graft survival can be useful in cases such as *ex-vivo* liver resection and autologous liver transplantation for end-stage hepatic alveolar echinococcosis. Till now, these results can’t be applied as a substitute for traditional liver transplantation. So, the next step is to utilize the new model for the transplantation of fully recellularized BELs. That’s why we developed our co-seeded grafts as a step toward the next tri-seeded grafts by adding cholangiocytes and then transplanting them in the orthotopic position, as we established in our latest model.

## Abbreviations

BELs, Bioengineered livers; rBELs, revascularized bioengineered livers; HCC, Hepatocellular carcinoma; NAFLD, Non-alcoholic fatty liver disease; ALD, Acute liver disease; ESLD, End stage liver disease; ECM, Extracellular matrix; ECs, Endothelial cells; PV, Portal vein; SHIVC, Suprahepatic inferior vena cava; HUVECs, Human umbilical vein endothelial cells; HG, Heparin-gelatin mixture; GFP, Green fluorescent protein; MSCs, Mesenchymal stem cells; TAA, Thioacetamide; iPSCs, Induced pluripotent stem cells; AFP, Alpha-fetoprotein; ALB, Albumin; GCR, Glucose consumption rate; IHIVC, Infrahepatic inferior vena cava; IVC, Inferior vena cava.
